# copMEM2: robust and scalable maximum exact match finding

**DOI:** 10.1093/bioinformatics/btad313

**Published:** 2023-05-12

**Authors:** Szymon Grabowski, Wojciech Bieniecki

**Affiliations:** Institute of Applied Computer Science, Lodz University of Technology, 18 Stefanowskiego Street, Lodz, Poland; Institute of Applied Computer Science, Lodz University of Technology, 18 Stefanowskiego Street, Lodz, Poland

## Abstract

**Summary:**

Finding Maximum Exact Matches, i.e. matches between two strings that cannot be further extended to the left or right, is a classic string problem with applications in genome-to-genome comparisons. The existing tools rarely explicitly address the problem of MEM finding for a pair of very similar genomes, which may be computationally challenging. We present copMEM2, a multithreaded implementation of its predecessor. Together with a few optimizations, including a carefully built predecessor query data structure and sort procedure selection, and taking care for highly similar data, copMEM2 allows to compute all MEMs of minimum length 50 between the human and mouse genomes in 59 s, using 10.40 GB of RAM and 12 threads, being at least a few times faster than its main contenders. On a pair of human genomes, hg18 and hg19, the results are 324 s and 16.57 GB, respectively.

**Availability and implementation:**

copMEM2 is available at https://github.com/wbieniec/copmem2.

## 1 Introduction

*Maximal exact matches* (MEMs) are exact matches between two strings (genomes) that cannot be further extended to the left or right. Their applications include seeding alignments of sequencing reads for genome assembly (Liu and Schmidt 2012), designating anchor points for genome-genome comparisons (Kurtz et al. 2004) and referential genome compression (Liu et al. 2017). Although the MEM finding problem can be solved in linear time using the suffix tree (Kurtz et al. 2004), this approach is memory hungry and can also be beaten in terms of practical speed. Alternatives include making use of a hash table (HT) for *k*-mers sampled from the reference (*R*) genome, applied in E-MEM (Khiste and Ilie 2015), sampling both the reference and the query (*Q*) in steps being relatively prime, as proposed in copMEM (Grabowski and Bieniecki 2019), and combining the copMEM approach with Bloom filtering the *k*-mers sampled from *Q*, introduced in bfMEM (Liu et al. 2019).

The last two cited works remain the state-of-the-art. copMEM is much faster in single-threaded comparisons, but lacks a multithreaded mode and uses significantly more memory than bfMEM. To address its shortcomings, we propose copMEM2, a multithreaded MEM finding tool, targeting the execution speed and reducing the memory, as well as incorporating an improvement to speed up its processing by orders of magnitude when the pair of genomes is highly similar.

## 2 Methods

The general architecture of the presented tool, copMEM2, resembles copMEM. A HT is built over regularly sampled *k*-mers from the reference genome, then substrings from the query genomes are found via HT, and finally left- and right-extended. As in other tools solving this problem, only MEMs of length at least runtime-specified *L* are sent to the output. One of the novelties of the presented software is that all main phases, i.e. HT construction and match finding, extending, filtering and sorting, are performed in parallel, using multiple threads.

Apart from the parallel processing, copMEM2 was optimized in a few ways.

The match sorting algorithm is no longer only std::sort (based on quick sort), but rather two radix sort variants, a most significant digit (MSD) one (https://github.com/voutcn/kxsort) and a least significant digit (LSD) radix sort on the most significant 6 bytes in match tuples (i.e. 6 digit passes) followed with std::sort in remaining buckets (this works faster than LSD radix sort over the whole keys), which, together with std::sort, are selected for different cases.

The matches are ordered by their starting position in *Q*, and then by their starting position in *R*. One option is to find all matches for each successive sequence (chromosome) in *Q* and then sort them appropriately, but for small *L* and large chromosomes it may require several gigabytes of memory. Our implemented solution is to sort matches in blocks (of $2^{21}$ elements), with large enough overlaps between blocks to prevent incorrect order on block boundaries. In the unlikely case of having the overlap too small, a slower and more memory demanding rescue procedure is called.

Mapping the starting match position to a sequence in *R*, which requires a predecessor query, no longer uses std::lower_bound, but builds a static data structure with predecessor answers for regularly sampled positions, in gaps dictated by the shortest reference sequence length. The preprocessing cost is more than offset with constant-time query handling.

We also noted that copMEM (i.e. the predecessor of the currently presented algorithm), similarly to most other competitors, is slow if *R* and *Q* genomes are very similar (e.g. two individuals of the same species). According to our knowledge, the only MEM finding tool which directly addresses this problem is E-MEM2 (Portes de Cerqueira Cesar 2020) (https://ir.lib.uwo.ca/etd/6837/), which is not publicly available. We (essentially) implemented the idea from E-MEM2, which is to discard a MEM (of length at least *L*) if it is fully contained in a MEM found at a previously sampled position in *Q*. In this way the number of accesses to *R* is hugely reduced in highly similar genomes, which avoids a great number of possibly costly match extensions.

Found MEMs are stored in internal buffers (one per thread) and occasionally dumped to temporary disk files; those files are merged into one at the end. This simple idea may reduce the memory usage a few times, if the total number of MEMs is huge (e.g. for L=50).

The sampling parameters for *R* and *Q* are $k_{1}$ and $k_{2}$, respectively, and they always differ by 1 in copMEM. This is relaxed in copMEM2: $k_{1}$ may be increased as much as possible to have the condition $k_{1}\cdot k_{2}\leq L-K+1$, where *K* is the seed size, still fulfilled, and $k_{1}$ and $k_{2}$ coprime. Note this helps, e.g., for L=80, where *K* is set to 44, as copMEM sets $\left(k_{1},k_{2}\right)$ to (6,5), while copMEM2 to (7,5), and even more for (L=132,K=44), where the parameters are changed from (9,8) to (11,8).

Additionally, we included a memory-frugal mode (-mf), in which a smaller HT and smaller buffers imply reduced memory usage, for a little penalty in performance.

Below we present in detail only three of the ideas incorporated in copMEM2; descriptions of the other ones can be found in the supplementary material.

### 2.1 Dealing with large match lists

Taming the memory usage and computational time for ordering the matches before they are dumped to disk is one of the challenges we faced in copMEM2.

Found matches, in the form of triples of integers, are appended to a list. Such a list has to be created for each sequence from *Q* and should finally be sorted and pushed to a file before memory is freed. However, the lists can be very long and occupy several GBs in the memory, especially for a small *L* and multiple threads running in parallel.

Our MEM-finding algorithm does not guarantee that matches will be found sequentially nor that they will be unique. Nevertheless, we have noticed that the obtained list is partially sorted, i.e. it is unlikely that a MEM on the unsorted list is far from its final (correct) location, so there is no need to keep the entire list in memory.

The procedure does the following:

- it appends a match to the match list *Matches*,
- if the current size of *Matches* reaches MATCH_BLOCK (set to $2^{21}$) elements, it
  - sorts the list *Matches*,
  - dumps 3/4 of MATCH_BLOCK elements from the beginning of *Matches*,
  - removes dumped elements from *Matches* (up to one-fourth remains).

An illustrating example is given in the supplementary material.

### 2.2 Match sorting strategy

In copMEM the matches were sorted with std::sort. In copMEM2, they are sorted with three sorting algorithms: std::sort (which is based on quick sort, combined with insertion sort for small subsets), the kxsort implementation of MSD radix sort (https://github.com/voutcn/kxsort), and our own 6-digit (i.e. 6-byte) LSD radix sort implementation followed with std::sort to order properly the remaining (usually small) intervals.

The details of our solution were worked out based on the following observations: (i) std::sort is a good choice for small match subsets, (ii) kxsort is more memory frugal than our LSD radix sort, and (iii) the hybrid of 6-pass LSD radix sort and std::sort may be the fastest choice, unless the data don’t fit the L3 cache, which indeed is a problem in a multiple thread scenario. In particular, we apply std::sort for collections up to MATCHES_SORT_T1 (default value: 1024) items, otherwise one of the other radix sort algorithms is used. When the number of working threads is up to 2 and $|R|<2^{32}$, we use our LSD radix sort based variant and in the remaining cases the kxsort algorithm is applied.

### 2.3 Fast sequence search

Mapping the match position to a sequence in *R* it belongs to, which requires a predecessor query, no longer uses std::lower_bound in copMEM2, but builds a static predecessor query data structure, which stores predecessor answers for regularly sampled positions, in gaps dictated by the shortest reference sequence length. The preprocessing cost is more than offset by constant-time query handling. The position of each found MEM, which requires referring to the HT entries, is absolute, but the textual output requires storing the match locations relatively to the beginning of their corresponding sequences (together with their names) in *R* and *Q* genomes. In copMEM (i.e., our old software) the positions of successive sequences from *R* were stored in a sorted array and each MEM required finding the rightmost sequence from *R*, whose start position was not greater than the MEM’s start position. This translated to a binary search procedure and was implemented with an invocation of the C++ STL’s lower_bound algorithm. In copMEM2 we find the shortest sequence from *R*, let us denote its length by $\ell_{\min}$, and sample *R* in regular steps of $\ell_{\min}$, storing the beginning of the *R*’s sequence each sampled position belongs to (plus the reference to its name) in array *Beg* of length $\left\lceil (|R|+0.5)/\ell_{\min}\right\rceil$.

**Figure 1.**
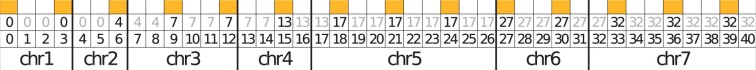
Example of the static predecessor query data structure in copMEM2.

Let us illustrate it with a simple example (cf. Fig. 1). The initial array of sequences is {(chr1, 0), (chr2, 4), (chr3, 7), (chr4, 13), (chr5, 17), (chr6, 27), (chr7, 32)}. The shortest sequence is chr2, whose length is 3. For this reason, we sample *R* with step 3. If, e.g. a match starts at position 17 in *R*, we access Beg[⌊17/3⌋]=Beg[5]=(chr4,13) and its successor (if exists), i.e. Beg[6]=(chr5,17). As 17≥17, we conclude that the match starts in sequence chr5. Let us have another example, with a match starting at position 14 in *R*. We access Beg[⌊14/3⌋]=Beg[4]=(chr3,7). Its successor is Beg[5]=(chr4,13). As 14≥13, we conclude that the match starts in chr4. Note also that a match starting at position 12 would belong to chr3 (as 12<13). In the actual implementation, to avoid divisions, we replace the computed step $\ell_{\min}$ with the largest power of 2 not greater than $\ell_{\min}$ (i.e. 3 would be replaced with 2 in this example).

## 3 Results

To evaluate the performance of copMEM2, we chose pairs of real multi-FASTA datasets used in earlier works, plus a pair of human genomes, hg18 and hg19. The Supplementary Material contains the dataset URLs and characteristics. The tests were conducted on Intel Core i9-10940X (14 cores) 3.3 GHz CPU, 128 GB of DDR4-RAM (CL 16, clocked at 2666 MHz), and a fast SSD (ADATA 2 TB M.2 PCIe NVMe XPG SX8200 Pro), running Linux (Debian 11) OS. All codes were written in C++ and compiled with gcc 10.2.1 -O3.

The presented timings, are median times of 3 runs in a particular setting (pair of datasets, chosen *L*, and possibly the number of threads). Before each run, the RAM memory of the linux machine was filled with a dummy value, to flush disk caches. In other words, all tests were run with a “cold cache.”

MEM finding times and RAM usages are given in Table 1. Usually 12 threads were selected (-t 12), but to compare copMEM2 with copMEM, it is also run in a single-threaded mode (-t 1). We were not able to obtain E-MEM2 to test it, but according to the results presented in (Portes de Cerqueira Cesar 2020, Chap. 4), it would not be competitive to copMEM2, even in the hg18 vs hg19 test.

**Table 1.** MEM results.^a^

MEM alg.	*H. sapiens* vs *M. musculus*				*H. sapiens* vs *P. troglodytes*				*T. aestivum* vs *T. durum*				*hg18* vs *hg19*			
	L=50		L=200		L=50		L=200		L=50		L=200		L=50		L=200	
	Time	RAM	Time	RAM	Time	RAM	Time	RAM	Time	RAM	Time	RAM	Time	RAM	Time	RAM
E-MEM -t 1	1866.3	9.24	497.7	**2.54**	4973.0	12.44	806.9	**2.69**	715.0	13.55	454.1	**3.85**	—	—	141 732.0	**2.62**
copMEM	907.5	17.99	23.5	6.52	3262.9	44.64	60.2	6.62	362.1	18.96	47.8	9.81	—	—	121 265.0	6.65
bfMEM -t 1	1815.4	12.03	298.1	4.91	6509.9	39.34	312.3	5.17	753.4	15.81	376.2	7.85	—	—	304 931.0	5.75
copMEM2 -t 1	311.9	8.14	25.3	6.53	1211.9	**8.19**	44.8	6.61	179.6	10.65	47.2	8.46	2719.2	24.77	56.8	6.57
E-MEM -t 12	1170.9	9.24	103.4	**2.54**	3970.0	12.44	147.2	**2.69**	251.8	13.55	118.3	**3.85**	148 622.0	20.89	19 346.0	**2.62**
bfMEM -t 12	517.0	**7.27**	33.2	6.57	1931.1	23.08	36.8	7.00	110.1	15.82	50.3	7.85	97 505.3	**13.79**	51 779.0	10.27
copMEM2 -t 12	**59.0**	10.40	9.4	8.09	**225.0**	10.47	**11.5**	8.47	**31.0**	11.34	13.1	9.11	**323.9**	16.57	**18.4**	8.35
copMEM2 -t 12 -mf	63.5	9.18	**8.8**	7.01	235.3	9.25	14.9	7.37	34.2	**10.13**	**12.1**	7.92	329.0	15.51	27.4	7.23

a Times in seconds, memory (RAM) usages in GBs (*G* = $10^{9}$).

Test platform: Intel Core i9-10940X (14 cores) 3.3 GHz CPU, 128 GB of DDR4-RAM (2666 MHz, CL 16) and a fast SSD (ADATA 2 TB M.2 PCIe NVMe XPG SX8200 Pro), running Linux (Debian 11) OS. All codes written in C++ and compiled with gcc 10.2.1 -O3. The copMEM2 values of $\left(k_{1},k_{2},K\right)$ for L=50 (resp. L=200) are (5,3,36) (resp. (13,11,56)) in the default mode and (5,3,36) (resp. (13,12,44)) in the -mf mode. The bfMEM times and max memory usages include match ordering postprocessing.

All genus names are expanded in Table 1 of the Supplementary Material.

In the single-threaded experiments copMEM2 is a clear winner in speed, usually beating its predecessor by a factor of about 2–3 for L=50, but rather slightly for L=200, where there are not so many matches to process. The gap grows to at least three orders of magnitude in the case of highly similar genomes (hg18 and hg19). Switching to 12 threads boosts copMEM2 by a factor from 5.3 to 8.4 (L=50) and from 2.7 to 3.9 (L=200). In the latter case, the speedup is smaller than offered by E-MEM and bfMEM, but still copMEM2 is always more than three times faster, and the gap is greater for L=50. In memory usage, copMEM2 -t 1 is significantly less demanding than copMEM for L=50, but the difference is small for L=200. The memory-frugal mode (-mf) reduces the RAM usage by more than 1 GB for usually up to 10% longer processing times. Overall, we can say that copMEM2 working in a multithreaded mode, tends to be a few times faster (or orders of magnitudes faster in case of highly similar genomes) than its contenders, while spending a comparable amount of memory (although E-MEM is clearly more succinct for large *L*).

In the experiments presented in Table 2, pairs of canine and feline genomes were used. Three MEM-finding tools were used, run only in the 12-thread mode. copMEM2 is a clear winner in speed, beating the second bfMEM by a factor from 2.7 (*Felis catus*, L=100) to 5.9 (*Canis familiaris*, L=80). Its advantage in speed over E-MEM is often about an order of magnitude. In memory usage, E-MEM is the most frugal while copMEM2 and bfMEM use roughly a similar amount of memory. This experiment suggests that these two pairs of genomes are not as similar as the human ones, where the gap in processing times between the tools was a few orders of magnitude.

**Table 2.** MEM results on pairs of same-species datasets.^a^

	Canis familiaris (canFam6 vs canFam4)						Felis catus (felCat9 vs felCat8)					
Tool	L=80		L=100		L=200		L=80		L=100		L=200	
	Time	RAM	Time	RAM	Time	RAM	Time	RAM	Time	RAM	Time	RAM
E-MEM -t 12	751.9	3.86	350.2	3.12	139.6	2.03	2140.9	10.05	686.6	3.36	160.8	2.20
bfMEM -t 12	360.9	9.30	144.8	8.51	47.0	6.88	901.1	14.91	323.4	10.04	49.7	8.88
copMEM2 -t 12	61.0	7.64	36.8	7.48	12.2	6.75	262.2	18.47	119.9	9.87	18.0	7.75

a 1 GB = $10^{9}$ bytes.

*Note*: The bfMEM times and max memory usages include match ordering postprocessing.

In the last experiment, we evaluate the impact of the number of working threads on the three MEM-finding tools. We took two cases, in which *L* was set to 300 (large output) and 50 (small output).

**Figure 2.**
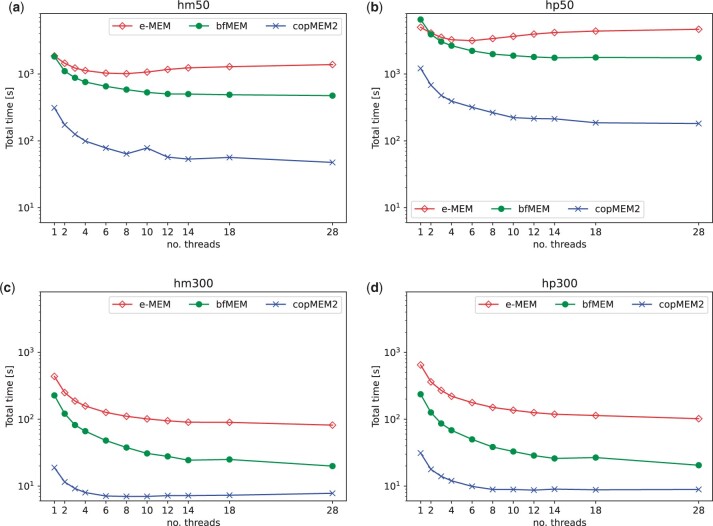
The varying performance of the MEM-finding tools with a growing number of threads. (a) Speedup with 14 threads compared to 1 thread, for E-MEM, bfMEM, copMEM2: 1.35, 3.85, 6.55. (b) Speedup with 14 threads compared to 1 thread, for E-MEM, bfMEM, copMEM2: 1.07, 3.73, 6.68. (c) Speedup with 14 threads compared to 1 thread, for E-MEM, bfMEM, copMEM2: 5.36, 11.42, 2.43. (d) Speedup with 14 threads compared to 1 thread, for E-MEM, bfMEM, copMEM2: 6.37, 11.50, 3.50.

Figure 2 shows that for L=50 E-MEM’s parallelization capabilities are not on a par with bfMEM and copMEM2, and its peak performance is for 6 or 8 threads. For small *L* copMEM2 is about 5.7–5.8 times faster with 14 threads compared to single-threaded runs. For bfMEM the corresponding speedup factors are about 3.7. Note however that bfMEM is a 2-phase tool: after its main processing it requires a postprocessing procedure (which is implemented as a single-threaded one) to order properly the output. For this reason, its overall parallelization in large output scenarios is not so impressive. Without the postprocessing routine, bfMEM’s speedup with 14 threads is close to 7-fold.

For L=300 bfMEM wins in the parallelization category. Still copMEM2 is the fastest software (for 14 threads it is 3 times faster than bfMEM). The bottlenecks are probably the reading time of the input files, the limited possibilities of prefetching and parallel access to memory.

## 4 Conclusion

Parallelism and algorithmic engineering are powerful tools aiming at software optimization, especially targeting modern hardware. In copMEM2, our proposed MEM-finding solution, we combined a number of ideas augmenting the original copMEM scheme, to obtain significant speedups and memory usage reductions. In particular, copMEM2 running with 12 threads on a multicore CPU is faster than its main competitors roughly by a factor between 3 and more than 10.

## Supplementary Material

btad313_Supplementary_Data

## Data Availability

The data underlying this article are available in multiple repositories, with their URLs given in Sec. 1 of the supplementary material.
