# Supplementary material for: copMEM2: robust and scalable maximum exact match finding
Source: Bioinformatics. 2023 May 12;39(5):btad313. doi: 10.1093/bioinformatics/btad313 (PMC10209524; doi:10.1093/bioinformatics/btad313)
Supplement: btad313_Supplementary_Data [file btad313_supplementary_data.pdf]

Supplementary Material to: "copMEM2: Robust and scalable maximum exact match finding"

Szymon Grabowski and Wojciech Bieniecki

April 25, 2023

1 Used datasets

Please download the following datasets and extract them (each to a separate directory). If an archive contains multiple files, they have to be concatenated to be used as one of the two input files for copMEM2 (or other MEM tools in our test procedure). Note that some of the URLs below have artifical linebreaks, so use them carefully.

1. Homo sapiens
   `https://hgdownload.cse.ucsc.edu/goldenPath/hg19/bigZips/chromFa.tar.gz`
   (ca. 900 MB of gzipped size)
   `https://hgdownload.cse.ucsc.edu/goldenPath/hg18/chromosomes/`
   `https://hgdownload.cse.ucsc.edu/goldenPath/hg19/chromosomes/`

2. Mus musculus
   `https://hgdownload.cse.ucsc.edu/goldenPath/mm10/bigZips/chromFa.tar.gz`
   (ca. 830 MB of gzipped size)

3. Pan troglodytes
   `https://hgdownload.cse.ucsc.edu/goldenPath/panTro3/bigZips/panTro3.fa.gz`
   (ca. 900 MB of gzipped size)

4. Triticum aestivum
   `https://ftp.ensemblgenomes.org/pub/plants/release-22/fasta/triticum_aestivum/dna/`
   `Triticum_aestivum.IWGSP1.22.dna.genome.fa.gz`
   (ca. 1.3 GB of gzipped size)

5. Triticum durum
   `https://urgi.versailles.inra.fr/download/iwgsc/TGAC_WGS_assemblies_of_other_wheat_`
   `species/TGAC_WGS_durum_v1.fasta.gz`
   (ca. 970 MB of gzipped size)

6. Canis familiaris 6 (Dog10K_Boxer_Tasha/canFam6)
   `https://hgdownload.soe.ucsc.edu/goldenPath/canFam6/bigZips/canFam6.fa.gz`
   (725 MB of gzipped size)

7. Canis familiaris 4 (UU_Cfam_GSD_1.0/canFam4)
   `https://hgdownload.soe.ucsc.edu/goldenPath/canFam4/bigZips/canFam4.fa.gz`
   (771 MB of gzipped size)

8. Cat #1 Felis catus 9.0
   `https://hgdownload.soe.ucsc.edu/goldenPath/felCat9/bigZips/felCat9.fa.gz`
   (774 MB of gzipped size)

9. Cat #2 Felis catus 8.0
   `https://hgdownload.soe.ucsc.edu/goldenPath/felCat8/bigZips/felCat8.fa.gz`
   (816 MB of gzipped size)

Basic characteristics of the datasets are presented in Table 1. The experiments comprise the following pairs of datasets (see the column 'Index'), where the first index is the reference and the second one the query genome: 01–03, 01–04, 05–06, 01–02, 07–08, 09–10.

Table 1: Datasets used in the experiments

Index	Dataset	Size (MB)	Sequences
01	*Homo sapiens* (human, hg19)	3,137	93
02	*Homo sapiens* (human, hg18)	3,137	49
03	*Mus musculus* (mouse)	2,731	66
04	*Pan troglodytes* (chimp)	3,218	24,132
05	*Triticum aestivum* (common wheat)	4,391	731,921
06	*Triticum durum* (durum wheat)	3,229	5,671,204
07	Dog #1 - canFam6	2,249	147
08	Dog #2 - canFam4	2,414	2,198
09	Cat #1 - felCat9	2,453	4,508
10	Cat #2 - felCat8	2,584	267,625

2 How to run copMEM2

Assuming that the input genomes are named hum.all.fa and panTro3.fa, where the former is the reference and the latter the query genome, an exemplary minimal command line may look like:

```
./copmem2 -o hp-100.txt hum.all.fa panTro3.fa > hp-100.log
```

where `-o` specifies the output file.

For this command line the minimum MEM length is set by default to 100, although `-l` followed by number may be added. Currently, $L = 50$ is the minimum value handled by copMEM2.

Additionally, copMEM2 is capable of finding reverse-complement matches with the switch `-r`:

```
./copmem2 -r -o hp-300-r.txt -l 300 hum.all.fa panTro3.fa > hp-300-r.log
```

or both forward and reverse-complement matches with the switch `-b`:

```
./copmem2 -b -o hp-300-b.txt -l 300 hum.all.fa panTro3.fa > hp-300-b.log
```

Additional useful parameters:

`-mf` - switches to the memory-frugal mode. It reduces the number of bits holding the hash to 28 and sizes of the text buffers for the output generation.

`-t 4` - runs the program in the multithreaded mode (i.e., 4 working threads are used in this example). The passed value is the maximum number of threads the program can use while running, but the number of working threads is limited by the number of sequences in the query dataset. A maximum of 64 threads can be set.

3 Configuration

In order to thoroughly examine the properties of the software, it is possible to set some parameters in runtime, and rest of them even before the compilation phase. The change requires interference in the source code copmem2.cpp and makefile.

3.1 Tuning runtime parameters

The list of other parameters is available after `./copmem2 -h`. Those parameters may be used as diagnostic and for tuning the application in terms of speed for some datasets, yet are not normally needed.

Event logging level (thanks to these switches, it is possible to mute all messages or show all messages and parameters of the processed files):

- `-q` - quiet mode. No screen output,
- `-v` - verbose mode. Display all runtime details.

Output generation:

- `-r` – compute only reverse-complement matches,
- `-b` – compute forward and reverse-complement matches,
- `-ilb` – ignore lowercase bases. Not used by default (i.e., lowercase bases are NOT ignored). When set, all lowercase symbols will be treated as N.

Tuning parameters:

- `-H n` – changes hash function. Five functions have been implemented. 1: maRushPrime1HashSimplified (default), 2: xxhash32, 3: xxhash64, 4: metroHash64, 5: cityHash64,
- `-K n` – changes the number of symbols used for Hash calculation to n. Default K is 44 but it also depends on L,
- `-hash_bits n` – manually set a length of the hash in bits which affects the size of Hash Table. Implemented values are 28, 29, 30,
- `-k1 n` – manually set k_1. Use with -k2 option,
- `-k2 n` – manually set k_2. Use with -k2 option, k_1 and k_2 should be coprimes,
- `-e` – forces $k_2 = 1$, which is similar to E-MEM,
- `-fbr 1|2` – manually force big Ref – long datatypes for processing. 1 – big, 2 – huge. It affects Hash Table and output structures sizes (see Sec. 5.1),
- `-lm n` – long MEM threshold. Default is 4096. Its change may affect the performance when comparing similar sets, e.g. hg18-hg19 (see Sec. 5.5),
- `-multi1 n` – manually set a buffer size for HT creation. Range: 64–512. Should be a power of two. Newer CPU may need to increase this value,
- `-multi2 n` – manually set a buffer size for HT creation. Notes same as above,
- `-multi n` – manually set a buffer size for processing query. Notes same as above.

3.2 Compilation parameters

Some parameters cannot be variables, as this would adversely affect the performance of the program. For this reason, it is not possible to use them on the command line. Nevertheless, that can be changed during compilation.

In CopMEM2.cpp one can find some preprocessor definitions:

- `#define radixsort 1` – enables LSD Radix sort. If you set it to 0, std::sort is invoked and -1 disables all sorting (may produce false results). For results, see Sec. 4.

- `#define stdfmt 0` – enables fmt library for numbers to strings conversion. If you want std::string methods to be called instead, set this value to 1.
- `#define predsv 1` – turns on fast search for beginning of the sequence. If you set the value to 0, `std::lowerbound` method will be used.
- `#define dumptimer 0` – disables measurements and displaying of times used for sorting and formatting found MEMs before sorting. If you set the value to 1, these benchmarks will be shown. Note that frequently switching the timers slows down the entire process (see Sec. 4).

In makefile there exist some gcc compile switches:

- `-march=native` – makes the program run faster on some machines, but makes the code non-portable. In addition, it may clash with valgrind,
- `-funroll-loops` – further optimization for speed,
- `-O3` – yet another optimization for speed,
- `-pthread` – enables multithreading.

Other parameters that may be interesting for benchmarking are set as constant identifiers, also in CopMEM2.cpp.

- `constexpr int MAX_THREADS = 64` – limits the number of used threads. You may change it.
- `constexpr uint32_t MAX_MULTI = 512` – limits the size of the array used to prefetch. In case of a better CPU, it may be increased.
- `constexpr size_t DEF_MATCH_BLOCK_SIZE = 1 << 21` – the maximum number of elements for partial sorting. See sec. 5.4.
- `constexpr size_t MAX_BLOCK_SIZE = 1ULL << 31` – the maximum number of elements for full (emergency) sorting.
- `constexpr size_t MATCHES_SORT_T1 = 1ULL << 10` – the maximum number of elements in the array that are sorted with std::sort.
- `size_t MATCHES_BUFFER_SIZE = 1ULL << 24` – the maximum buffer size for formatting matches as a string before flushing them to disk in the query phase.

4 Benchmarking

All tests were performed in a Debian 11 environment. The `time` program was used to measure execution time and memory consumption. Listing 1 shows a script that tests E-MEM, bfMEM and copMEM2.

Listing 1: benchmarks.sh – exemplary linux script for testing competing software

```
#!/bin/bash
MemFill="./memoryfill 118G"

R="../datasets/hum.all.fa"
Q="../datasets/panTro3.fa"
TST="hp"

for pass in a b c
do
 for L in 200 100 80
```

```
 do
  for T in 8 4 1
  do
   echo "Test =" $TST "  Pass =" $pass "  L =" $L "  Threads =" $T
   # E-MEM
   eval $MemFill
   /usr/bin/time --verbose ../progs/e-mem -n -l $L -t $T $R $Q >
       $TST-$L-$T-$pass.txt
   rm $TST-$L-$T-$pass.txt

   # BF-MEM
   eval $MemFill
   /usr/bin/time --verbose ../progs/bfmem -r $R -q $Q -o $TST-$L-$T-bfmem.txt
       -l $L -t $T
   /usr/bin/time --verbose ../progs/formatconvert $TST-$L-$T-bfmem.txt
       $TST-$L-$T-$pass.txt
   rm $TST-$L-$T-bfmem.txt
   rm $TST-$L-$T-$pass.txt

   # COPMEM2
   eval $MemFill
   /usr/bin/time --verbose ./copmem2 -t $T -l $L -o $TST-$L-$T-$pass.txt $R $Q
   rm $TST-$L-$T-$pass.txt
  done
 done
done
```

The script is most conveniently run in the background with the nohup command.
`$ nohup ./benchmarks.sh > benchmarks.log &`
Explanations to the sample script:

- the sample script tests the hum and panTro3 datasets for minimum values $L = 200$, 100 and 80 for 1, 4 and 8 threads. Each test is performed 3 times (for the purposes of this article, we calculated the median of three measurements);
- all programs are in the ../progs dir and the test files are in the ../datasets dir. Result files are created in the current dir and they are deleted after the timing;
- before each test, the main memory is filled with `memoryfill` program (attached to the project). This prevents caching of the input files in the RAM memory;
- E-MEM, unlike the others, does not have a parameter specifying the output file. Therefore, the command includes redirecting standard output to a file;
- processing with bfMEM requires two phases. The main program, bfMEM, searches for the MEMs, but it sorts the matches in a manner inconsistent with other programs. Therefore, it is necessary to use the supplementary program formatconvert, provided by the authors.

5 Technical description of the copMEM2

This section presents copMEM2 technicalities.

The processing involves several phases (cf. Fig. 1). Note that the general MEM finding mechanism, as well as most of the data structures, are preserved from v1. Elements introduced or changed in copMEM2 are pointed out below.

- `scanMultiFasta` The function scans the genome Q for the beginning of individual sequences, saved in a list (i.e., C++'s vector). Then this list is split into $nThreads$ lists (where $nThreads$ is the specified number of threads) of approximately equal size. Elements of each list are grouped into blocks that will be loaded into memory at once. A block may contain one or more sequences, but its size cannot exceed the length of the longest sequence.
- `readMultiFasta` The function loads the genome R into the memory. It creates a list of sequences, analogously for Q, but each item is augmented with extra data, the sequence name (i.e., the list contains 2-element tuples). Additionally, the function:
  - removes sequence names and whitespaces from the array, and aligns the data,
  - converts all non-ACGT symbols into a special character, and converts the symbols to uppercase.
- Creating a hash table of K-mers sampled from R with step k_1. In copMEM2 this phase may be performed using multiple threads.
- Processing of the query dataset and result generation. This phase includes scheduling portions of data for threads. Each thread reads a sequence, finds its matches (i.e., MEMs), and dumps the results, in a sorted form, into a textual file. At the end, all these temporary files are merged into one.

Below, some details of key structures and algorithms are revealed.

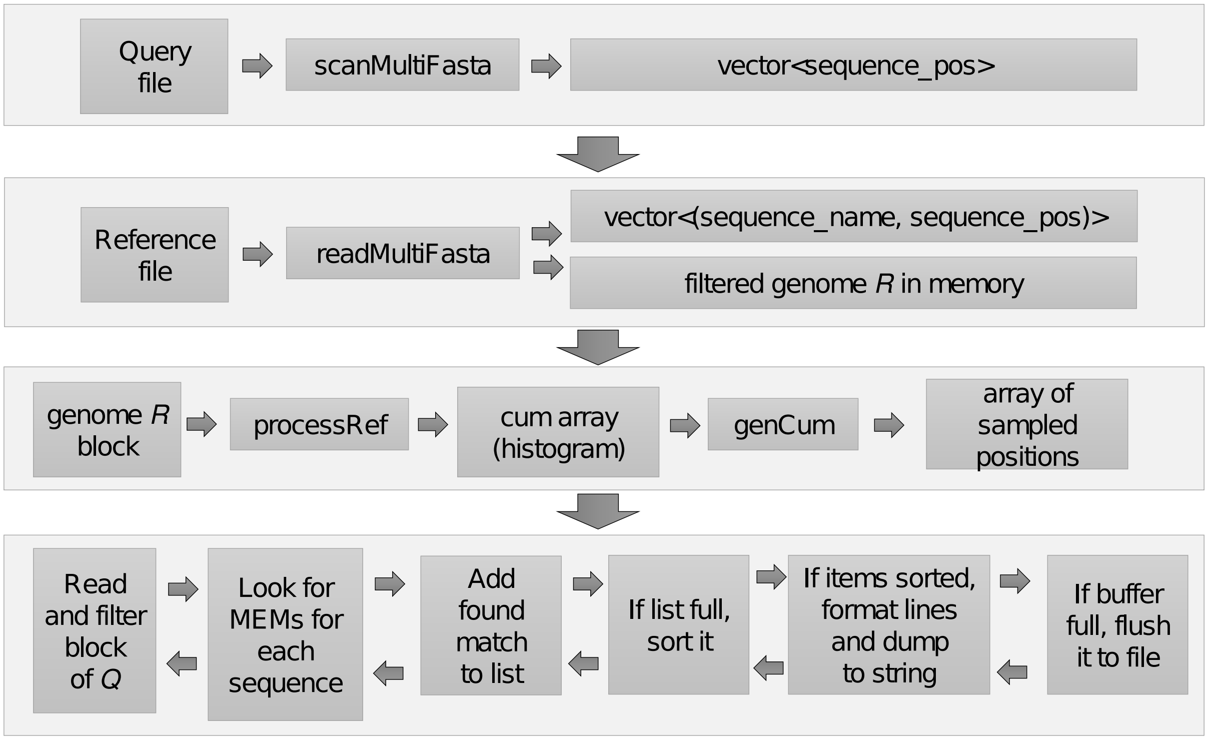

Figure 1: Data processing overview

5.1 Creation of a hash table

The concept of the hash table (HT) is the same as in copMEM. It will contain indices of R's suffixes sampled with step k_1, grouped by the hash of K-long prefix of each suffix. HT physically consists of two one-dimensional arrays:

- `cum` – the actual hash table, initialized with zeros. The elements of the array are unsigned 32-bit integers, if the size of R is at most $k_1 \cdot 4$ GB, or 64-bit ones otherwise. The number of elements in `cum` is 2^{29}, because 29 is the fixed number of hash bits.
- `sampledPos` – an array of occurrences. The elements are 32-bit integers if R's size is $4 \cdot k_1$ GB or less, otherwise 64-bit ones. There are $\lfloor (N - K + 1)/k_1 \rfloor + 2$ elements in the array (the extra 2 is added for technical reasons).

When the hashes of all $\lfloor (N-K+1)/k_1 \rfloor$ K-mers are computed (multiple threads can be used in this phase in copMEM2), and `cum` and `sampledPos` filled, each pair `cum[j]` and `cum[j+1]` will determine the interval of K-mers whose hash value is j. More precisely, assuming that `cum[j]` $\neq$ `cum[j+1]`, `sampledPos[cum[j]]` contains the index of the first K-mer whose hash value is j (if any), while `sampledPos[cum[j+1]]` contains the index of the first K-mer whose hash value is larger than j. Note that there may be no sampled K-mers with hash value j, but then the corresponding interval is empty.

The following improvements were implemented in copMEM2.

- The number of hash bits `HS` can be set to 29 (by default) or 28 (`-mf` mode). In the latter case, the array `cum` gets halved.
- When the size of the genome R is between 2^{32} and $k_1 \cdot 2^{32}$, we can still use 4-byte elements in the array `sampledPos`. In this case, the items are divided by k_1 while writing and multiplied by k_1 while retrieving. Note that k_1 is usually at least 4. Although the extra divisions and multiplications pose some overhead, this change seems to be beneficial on the overall.

Table 2 gives a couple of examples for the HT memory usage in copMEM and copMEM2. Note that the values of k_1 differ in the 5th and the 6th row; this is explained in Sec. 5.3.

Table 2: Illustration of the memory usage for the hash table, which is comprised of `cum` and `sampledPos` components, for copMEM and copMEM2 (default mode). The size of the reference sequence (R) is assumed to be 3 GB, 6 GB or 24 GB, respectively. 1 GB = 10^9 bytes.

Tool	$\lvert R\rvert$ [GB]	L	k_1	|`cum`| [GB]	|`sampledPos`| [GB]	HT mem. [GB]
copMEM	3	100	8	2.15	1.50	3.65
copMEM2	3	100	8	2.15	1.50	3.65
copMEM	6	100	8	4.29	6.00	10.29
copMEM2	6	100	8	4.29	3.00	7.29
copMEM	24	50	4	4.29	48.00	52.29
copMEM2	24	50	5	4.29	38.40	42.69
copMEM	24	100	8	2.15	24.00	26.15
copMEM2	24	100	8	2.15	12.00	14.15

5.2 Multithreading

In the considered programs, multithreaded processing has been implemented in a different way. E-MEM uses OpenMP. Additionally, the first phase of work, which is building a hash table, is single-threaded. bfMEM and copMEM2 use the low-level threaded programming available in C++. Both of these programs parallelize the MEM lookup in a similar manner, that is, the individual sequences in Q are distributed among the threads, such that the thread processes at least one whole sequence. Hence, there is a limitation in both programs that the maximum number of working threads must not exceed the number of sequences in Q (so in the extreme case of having a single sequence in Q, both bfMEM and copMEM2 will work in the single-threaded mode, no matter the chosen switches). We noticed that bfMEM reports the number of threads in an inaccurate

manner. For example, if we run it with a maximum number of 6 threads, the foreground thread spawns 6 worker threads and waits for their completion. In fact, the program then runs on 7 threads. (Neither E-MEM nor copMEM2 behave in this way.)

copMEM2 uses threads as follows.

- Reading and analyzing input files: this phase is performed serially (except for a subphase of replacing symbols `N` and `n`, which may involve 2 threads, with a minor benefit in speed).
- Creation of the hash table: the array containing the genome R is split evenly between threads for HT building.
- MEM finding phase. After analyzing Q, this genome is divided at sequence boundaries between threads so that each of them gets approximately the same portion of data. Each thread opens its (temporary) output file in which it will save the results in the target format.
- Merging temporary files: output files should be merged in the correct order. This is handled by the first thread just after it has finished processing of its portion of data. Meanwhile, other threads may be finishing their processing. This allows appending files partially in parallel with other threads still searching for matches.

This approach is quite frugal when it comes to using disk space. Details are given in Sec. 6.

5.3 Increasing k_1 when possible

In copMEM2, like in its predecessor, the sampling parameters k_1 and k_2 are relatively prime. In copMEM they are possibly close to $\sqrt{L-K+1}$; namely, k_1 is set to be the largest integer such that $k_1 \cdot (k_1 - 1) \leq L - K + 1$ and $k_2 = k_1 - 1$. This selection scheme is refined in copMEM2. We start as above, but then try to increase k_1 as much as possible, that is, to still have the condition $k_1 \cdot k_2 \leq L - K + 1$. The value of k_2 is unchanged.

Note that it helps only in several cases, in comparison to copMEM (but never hurts). For example, if $L = 80$, then K is set to 44 and while copMEM sets (k_1, k_2) to $(6, 5)$, in copMEM2 the sampling step over the genome R is increased to 7, i.e., $(k_1, k_2) = (7, 5)$. This reduces the memory usage for the hash table (namely, its `sampledPos` component), but also means that building the hash table can be sped up (optimistically) by a factor of 7/6. The gain is even greater for $(L = 132, K = 44)$, where copMEM sets (k_1, k_2) to $(9, 8)$ and copMEM2 to $(11, 8)$. There is no gain, e.g., for $(L = 100, K = 44)$, as both copMEM and copMEM2 set (k_1, k_2) to $(8, 7)$.

5.4 Dealing with large match lists

How copMEM2 handles large match lists is described in the main paper (Sec. 2.1), so the reader is advised to read it first. Now we only add an illustrating example.

Assume that `MATCH_BLOCK` = 16 and $Matches$, of length `MATCH_BLOCK`, is [1000, 2430, 432, 576, 246, 23, 102, 256, 334, 665, 234, 665, 823, 345, 43, 889], and the current element to add is 932. Then we:

- sort $Matches$ to obtain [23, 43, 102, 234, 246, 256, 334, 345, 432, 576, 665, 665, 823, 889, 1000, 2430],
- dump its first $3/4 \cdot 16 = 12$ elements to reduce the list to [823, 889, 1000, 2430],
- append 932 to $Matches$ to obtain [823, 889, 1000, 2430, 932].

In the unlucky case when the added element is too small (e.g., 800 in the example above, rather than 932), an emergency procedure is provided. In this procedure, the current thread must start from scratch and use a new value of `MATCH_BLOCK`, which is increased to 2^{31}. Note it does not affect the processing of the remaining threads. Note also that if `MATCH_BLOCK` is set (for safety) to a huge value, it does not mean that the space requirements of the tool grow correspondingly; it is much more likely that the current (even if large and problematic) sequence from Q is finished earlier and the thread's match list cleared.

5.5 Dealing with long matches

Another problem to be solved is finding matches in similar genomes, as in the case of the hg18-hg19 test. A substantial number of very long MEMs will be found in such tests. With scanning Q with some fixed increment, it is likely that the same MEM will be found multiple times despite a changing anchor. The other contenders cannot effectively handle such cases and search for the same match multiple times, deleting the duplicates only before formatting the results. This explains their long processing times for the pair of datasets hg18 and hg19. A mechanism to prevent this type of situation was designed in the (non-public) E-MEM2 software.

We (essentially) implemented the idea from E-MEM2, which is to discard a MEM (of length at least L) if it is fully contained in a MEM found at the previously sampled position in Q; in this way the number of accesses to R is hugely reduced in highly similar genomes, which avoids a great number of possibly costly match extensions.

More precisely, if a match is found whose length from the seed position to its right boundary is at least `LONG_MEM` (set to 4096 by default), newer matches are handled in a special way. Let us denote the mentioned long match by LM. We check in the current match if its right boundary is still within the span of LM and the difference between the positions of the seed in Q and its 'anchor' reference in R is still the same as such a difference for LM. Such a case means that at the current position we found exactly the same match as before (which is going to be of length at least `LONG_MEM`), therefore we can skip its (costly) extension.

5.6 Minor improvements

Using the fmt library

In copMEM2 we use the open-source formatting library `fmt` for creating formatted match lines (converting unsigned integers to strings). To our knowledge, it provides the fastest portable alternative to C stdio and C++ I/O streams. fmt is available at `https://fmt.dev`.

Variable prefix length (K)

In copMEM K has a fixed value (default 44, and the user could choose K from $\{36, 44, 56\}$). copMEM2 enables manual setting of K, from 32 to 96, as a multiple of 4. If K is not set by a parameter, the program will assign $K = 56$ for $L \geq 200$, $K = 44$ for $L \in \{80, \ldots, 199\}$ and 36 for a smaller L.

Memory frugal mode

In the introduced memory-frugal mode (-mf) the number of slots in the hash table is halved and the size of the buffer holding text-formatted output before dumping it to file is reduced from 2^{24} to 2^{22} bytes. Additionally, the seed size K is set a bit differently than in the default mode: to 44 if $L \geq 200$ and to 36 otherwise.

Table 3 shows the progress of copMEM development. The basic copMEM2 architecture is compared to the old version of our software (copMEM), and then is augmented with optimizations in match sorting (related to output generation), sequence search and printing the textual messages, to be sent as the output. Note that all these changes essentially improve the performance only when the number of MEMs is large, e.g., for small values of L in our experiments. The first two sets of columns, "copMEM" and "copMEM2, basic" demonstrate the impact of general changes in the (single-threaded) architecture of copMEM2. The large speedup, exceeding the factor of 2 in the hp50 experiment, is obtained mostly due to sorting the matches in blocks rather than in total. This more than offsets the extra I/O operations responsible for merging the output on disk. Also the match items, to be sorted, are stored more compactly in copMEM2, which helps both in speed and memory usage. Changing the sorting algorithm affects the overall performance only moderately, up to about 8%. On the other hand, the static predecessor query solution resulted in a bigger gain, up to about 16%. However, it comes at a cost of a slight increase of the memory usage. The improvement from using the fmt library is rather small and inconsistent.

6 Additional results

Table 3 shows the progress of copMEM development.

Table 3: Impact of copMEM2 modifications

Test	copMEM		copMEM2, basic		+opt. sort		+pred. search		+fmt	
	Time	RAM	Time	RAM	Time	RAM	Time	RAM	Time	RAM
hp300, $t = 1$	42.9	6.4	31.2	6.4	31.2	6.4	31.1	6.4	31.1	6.4
hp300, $t = 12$	—	—	10.0	8.2	9.1	8.2	8.7	8.2	8.8	8.2
tatd300, $t = 1$	39.1	9.3	34.3	8.1	32.8	8.1	33.8	8.2	32.3	8.2
tatd300, $t = 12$	—	—	12.4	8.7	10.9	8.7	11.7	8.8	10.7	8.8
hp50, $t = 1$	3263.8	44.6	1508.4	8.2	1398.2	8.2	1243.6	8.2	1231.2	8.2
hp50, $t = 12$	—	—	249.2	10.5	232.8	10.5	224.0	10.5	232.9	10.5
tatd50, $t = 1$	364.1	19.0	212.1	10.5	209.5	10.5	179.8	10.7	179.0	10.7
tatd50, $t = 12$	—	—	37.2	11.2	35.1	11.2	30.2	11.3	29.2	11.3

hp300 means human vs. chimp with $L = 300$ and tatd 50 is common wheat vs. durum wheat with $L = 50$.
Serial and 12 thread processing. Times in sec, memory (RAM) usages in GBs (G = 10^9).

The basic copMEM2 architecture is compared to the old version of our software (copMEM), and then is augmented with optimizations in match sorting (related to output generation), sequence search and printing the textual messages, to be sent as the output. Note that all these changes essentially improve the performance only when the number of MEMs is large, e.g., for small values of L in our experiments. The first two sets of columns, "copMEM" and "copMEM2, basic" demonstrate the impact of general changes in the (single-threaded) architecture of copMEM2. The large speedup, exceeding the factor of 2 in the hp50 experiment, is obtained mostly due to sorting the matches in blocks rather than in total. This more than offsets the extra I/O operations responsible for merging the output on disk. Also the match items, to be sorted, are stored more compactly in copMEM2, which helps both in speed and memory usage. Changing the sorting algorithm affects the overall performance only moderately, up to about 8%. On the other hand, the static predecessor query solution resulted in a bigger gain, up to about 16%. However, it comes at a cost of a slight increase of the memory usage. The improvement from using the fmt library is rather small and inconsistent.

More detailed information on testing the sorting process itself is presented in Table 4. The hm50 and hp50 cases were selected because they generate the longest output lists (requiring 25 GB and 78 GB space on the disk, respectively).

The compile options presented in Sec. 3 allow to switch between C++ std and radix sorting modes. It is also possible to enable sort time logging. Unfortunately, this mode incurs some overhead due to the extra instructions to resume and pause timers. The presented study shows that using a combined radix sort algorithm is almost twice faster than using std::sort.

In all the original papers presenting the MEM tools used in our comparison, the RAM consumption of a running process was tested, but not the auxiliary disk space. To our knowledge, E-MEM and copMEM are the only programs that do not use more disk space than needed to store their output files. However, E-MEM uses a less compact output format (runs of space characters instead of tabs), which sometimes translates to close to 3 times larger output space. bfMEM and copMEM2 (the latter only in a multithreaded mode) produce temporary files during their work. Table 5 presents the results of disk occupancy measurements for the tested programs.

Both copMEM and copMEM2 for 1 thread use only as much disk space as needed to write the output file. copMEM2 for two or more threads needs additional space, which results from the fact that the final output file is created from joining temporary files generated by individual threads and additional disk space is needed during copying. This extra disk space can roughly be estimated as $|output|/nThreads$ (although in the unlikely worst case the overhead may be as large as $|output|$).

Measurements for the hm80, hm50, hp80 and hp50 test cases suggest that bfMEM needs around $2 \cdot |output|$ of the disk space. The corresponding disk space for copMEM2 was around $1.5 \cdot |output|$ in the worst case (2

Table 4: Timings of sorting long MEM lists

			hm50 output size 25 827 MB		hp50 output size 78 850 MB	
sort strategy	mode	#threads	total time	time per thread	total time	time per thread
default	debug	1	318.38	39.58	1231.66	164.69
	release	1	310.74		1204.77	
std::sort	debug	1	347.32	72.17	1345.71	294.21
	release	1	341.17		1344.43	
default	debug	2	177.22	20.26	685.81	84.09
	release	2	173.42		674.99	
std::sort	debug	2	193.32	38.26	741.28	156.20
	release	2	190.16		743.93	
default	debug	6	79.53	7.07	331.37	29.39
	release	6	80.36		323.09	
std::sort	debug	6	88.33	14.34	355.88	58.19
	release	6	87.42		359.61	
default	debug	12	59.59	3.63	229.66	15.26
	release	12	58.93		220.63	
std::sort	debug	12	63.48	7.41	231.58	30.27
	release	12	63.30		236.37	

mode = debug means that $dumptimer = 1$ is set.
This option allows to measure sorting times, but at the same time gives some time overhead.
hm50 (resp. hp50) means human vs. mouse (resp. human vs. chimp) with $L = 50$.

threads) and only $1.12 \cdot |output|$ with 12 threads.

7 Referred software

- copMEM2
  `https://github.com/wbieniec/copmem2`
- copMEM
  `https://github.com/wbieniec/copmem`
- E-MEM
  `https://github.com/lucian-ilie/E-MEM`
- bfMEM
  `https://github.com/yuansliu/bfMEM`
- CityHash
  `https://github.com/google/cityhash`
- MetroHash
  `https://github.com/jandrewrogers/MetroHash`
- XXHash
  `https://github.com/Cyan4973/xxHash`
- MaRushPrime hashing
  `http://www.amsoftware.narod.ru/algo2.html`

Table 5: Disk usage for selected datasets, in megabytes

	hm80 (166 MB)	hp80 (9 593 MB)	hm50 (25 827 MB)	hp50 (78 850 MB)
copMEM	166	9 593	25 827	78 850
copMEM2 -t 1	166	9 593	25 827	78 850
copMEM2 -t 2	166	14 606	38 370	117 611
copMEM2 -t 6	177	11 477	30 623	92 873
copMEM2 -t 12	166	10 753	28 832	88 449
E-MEM -t 1	456	26 232	71 222	218 130
E-MEM -t 2	456	26 232	71 222	218 130
E-MEM -t 6	456	26 232	71 222	218 130
E-MEM -t 12	456	26 232	71 222	218 130
bfMEM -t 1	166	20 627	56 603	209 736
bfMEM -t 2	166	20 626	56 606	209 734
bfMEM -t 6	166	20 626	56 609	209 738
bfMEM -t 12	166	20 625	56 604	209 727

Usage of the working directory was calculated by `du -s` command run every 0.1 sec. while the program was operating. The size of output files for bfMEM, copMEM and copMEM2 are given in the header. The corresponding output files for E-MEM are, however, larger: of size 456 MB, 26 232 MB, 71 222 MB and 218 130 MB, respectively.

- kxsort
  `https://github.com/voutcn/kxsort`
- RadixSort
  `https://github.com/lemire/Code-used-on-Daniel-Lemire-s-blog/tree/master/2021/04/09`
- fmtlib::fmt
  `https://github.com/fmtlib/fmt`
